# Velocity storage mechanism drives a cerebellar clock for predictive eye velocity control

**DOI:** 10.1038/s41598-020-63641-0

**Published:** 2020-04-24

**Authors:** Shuntaro Miki, Kohei Urase, Robert Baker, Yutaka Hirata

**Affiliations:** 1 Department of Computer Science, Chubu University Graduate School of Engineering, Kasugai, 487-8501 Japan; 2 Department of Neuroscience, New York University Langone Medical Center, NY, 10016 NY USA; 3 Department of Robotic Science and Technology, Chubu University College of Engineering, Kasugai, 487-8501 Japan

**Keywords:** Cerebellum, Brainstem, Cerebellum, Reflexes

## Abstract

Predictive motor control is ubiquitously employed in animal kingdom to achieve rapid and precise motor action. In most vertebrates large, moving visual scenes induce an optokinetic response (OKR) control of eye movements to stabilize vision. In goldfish, the OKR was found to be predictive after a prolonged exposure to temporally periodic visual motion. A recent study showed the cerebellum necessary to acquire this predictive OKR (pOKR), but it remained unclear as to whether the cerebellum alone was sufficient. Herein we examined different fish species known to share the basic architecture of cerebellar neuronal circuitry for their ability to acquire pOKR. Carps were shown to acquire pOKR like goldfish while zebrafish and medaka did not, demonstrating the cerebellum alone not to be sufficient. Interestingly, those fish that acquired pOKR were found to exhibit long-lasting optokinetic after nystagmus (OKAN) as opposed to those that didn’t. To directly manipulate OKAN vestibular-neurectomy was performed in goldfish that severely shortened OKAN, but pOKR was acquired comparable to normal animals. These results suggest that the neuronal circuitry producing OKAN, known as the velocity storage mechanism (VSM), is required to acquire pOKR irrespective of OKAN duration. Taken together, we conclude that pOKR is acquired through recurrent cerebellum-brainstem parallel loops in which the cerebellum adjusts VSM signal flow and, in turn, receives appropriately timed eye velocity information to clock visual world motion.

## Introduction

**Figure 1:**
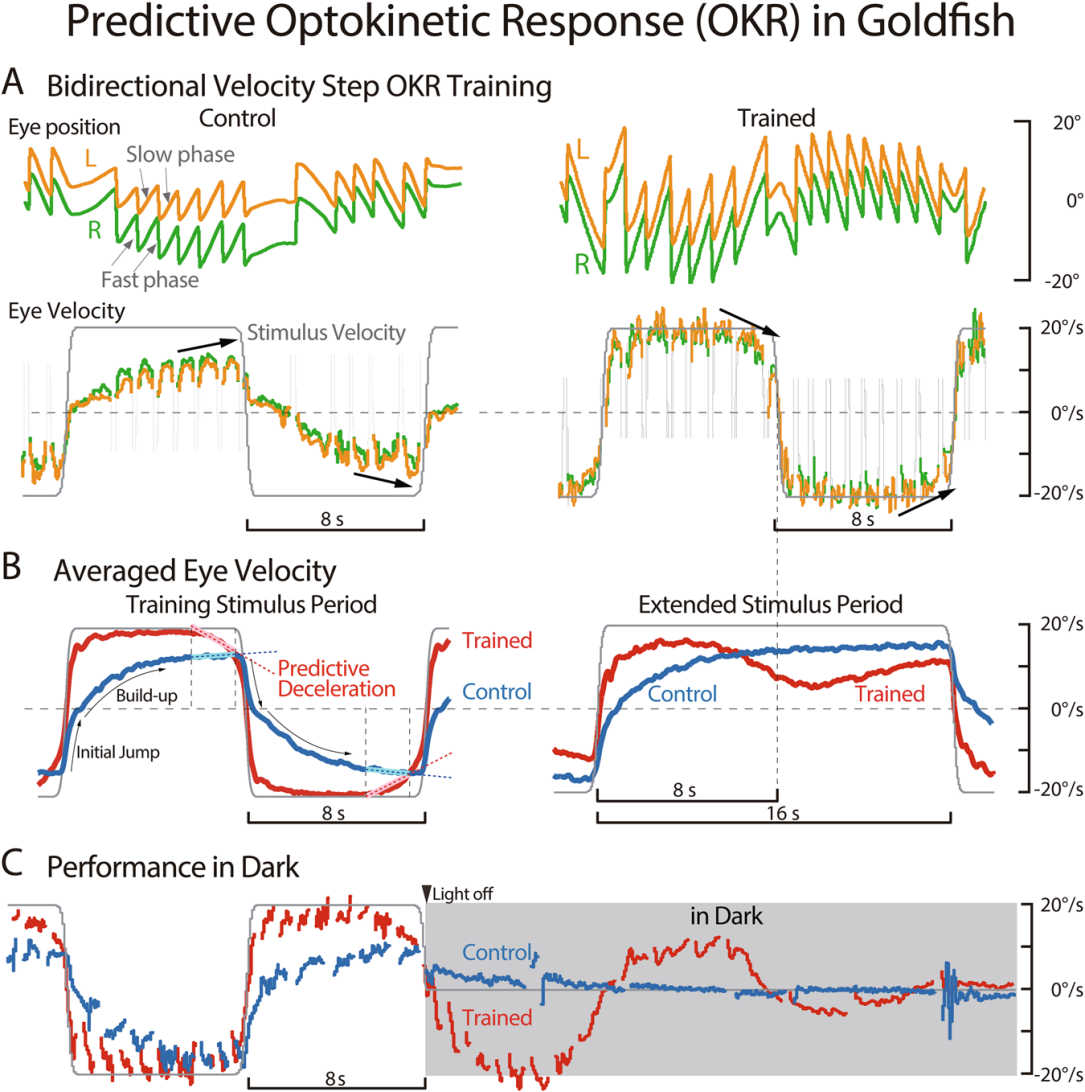
Predictive Optokinetic Response (OKR) in Goldfish. (**A**) Eye movement performance before (left, Control) and after (right, Trained) 180 min bidirectional velocity step training of a representative goldfish in response to a training stimulation whose half-period was 8 sec. Upper traces are left (L, orange) and right (R, green) eye positions consisting of slow and fast phases (gray arrows). Lower traces are slow phase eye velocity and visual stimulus velocity (gray). Fast phases in eye velocity are indicated by gray thin lines. Rightward (clockwise) and leftward (counter clockwise) directed eye positions are indicated by + and - of the ordinate, respectively. (**B**) Averaged eye velocity over 8 experiments before (blue, Control) and after (red, Trained) bidirectional velocity step training in response to the visual stimulation used for training (left, gray) and during extended period stimulation whose half-period was 16 sec (right, gray). (**C**) Performance of eye velocity in the dark before (blue) and just after (red) bidirectional velocity step training of the same fish as in A. Visual stimulation (gray) was turned off at the black down arrow head.

Predictive feedforward motor control is essential and ubiquitously employed in animal kingdom because biological sensory feedback delays are often too long to achieve rapid and precise motor behaviors. Visuomotor transformation systems have been studied to understand the underlying neuronal mechanisms achieving predictive motor controls. In many vertebrates, a large moving visual scene induces a reflexive eye movement called the optokinetic response (OKR) to stabilize the visual image on the retina. In natural environments, such large field visual motions are often evoked when animals move their head. During head motion, another reflexive eye movement called the vestibulo-ocular reflex (VOR) is primarily induced, and the eyes are counter-rotated in the orbits. However, the VOR is not perfectly compensatory, resulting in residual visual motion in the opposite direction to the head turn, thereby inducing the OKR. It has been shown in goldfish that the OKR system, that normally functions as a feedback control system^1^, acquires predictive feedforward control when a temporally periodic visual stimulation is presented^2,3^. Namely, OKR eye velocity induced by a large-field visual stimulation moving periodically to the left and right at a constant speed began to decrease before the visual stimulation switched direction (Fig. 1A Trained, black arrows; Fig. 1B Trained, Predictive Deceleration). When the visual stimulus was extended after the acquisition of this behavior, eye velocity decrease persisted even though the visual stimulation continued at a constant velocity (Fig. 1B Extended Stimulus Period, Trained). When the visual stimulus was not extended, but turned off, the eye velocity profile continued for several periods in the dark (Fig. 1C Trained). Periods that goldfish could predict ranged from 2 seconds to 128 seconds^2^.

The acquisition and maintenance of predictive OKR in goldfish was also shown to require an intact cerebellum^3^. Not only were Purkinje cells in the vestibulo-cerebellum found to modulate their firing patterns in a highly correlated manner with predictive components of OKR, but cerebellectomized fish lost both the ability to acquire and maintain the predictive components. Although these results suggested that the cerebellum was necessary for the acquisition of predictive OKR, other neuronal bases were not excluded from involvement in predictive OKR acquisition.

One of the distinctive characteristics of the cerebellum is its neuronal architecture that has been evolutionally well conserved from teleosts to primates^4–21^. We surmised that if the cerebellum alone is sufficient for the acquisition of predictive OKR, other species of fish than goldfish exhibiting particularly closely related cerebellar neuronal circuitry should be able to acquire the predictive oculomotor behavior. Therefore, we conducted behavioral experiments in four different fish species to test this hypothesis.

Our findings showed that like goldfish, carp acquired predictive oculomotor behavior while medaka and zebrafish did not. We noted that the two species acquiring predictive OKR exhibited robust optokinetic after nystagmus (OKAN), a behavior shown to be a manifestation of the velocity storage mechanism (VSM). By contrast, the OKAN of zebrafish and medaka was extremely short and they did not acquire predictive OKR. Since we observed that goldfish and carp with longer OKAN durations did not necessarily acquire predictive OKR better, we examined vestibular-neurectomized goldfish whose OKAN was severely shortened. Interestingly, predictive OKR was comparable to normal animals. Together these results suggest that the cerebellum alone is not sufficient to acquire predictive OKR. Instead brainstem-cerebellar loops involving a velocity storage mechanism is required, and irrespective of VSM capacity.

## Methods

### General procedures

Animals tested were goldfish (*Carassius auratus*), zebrafish (*Danio rerio*), medaka (*Oryzias latipes*), and Carp (*Cyprinus carpio*). Fish were obtained from an authorized supplier (Meitosuien Co., Ltd., Japan). In the laboratory, all fish were maintained at 26 °C on a 12 h light/dark cycle with the water quality monitored. Procedures for animal preparation were adopted from those previously described (Goldfish^2,22–24^, zebrafish and medaka^19,25^). Briefly, goldfish and carps of both sexes, sized 12 ~ 15 cm in length, were restrained by fitting the mouth onto a tapered tube and holding the body, wrapped in gauze for protection, in a plexiglass rigging lined by moistened sponges. The circular tank, 30 cm in diameter, had untextured white walls that acted as a blank background for the visual stimulus. Aerated water, at 26 °C, was passed over the gills for ventilation. Adult zebrafish and medaka of both sexes were restrained by wrapping with a sponge and fixated at the center of the 10.5 cm circular water tank that had untextured dark gray walls and acted as a blank background for the visual stimulus. Aerated water at 26 °C was filled in the water tank and maintained the zebrafish and medaka healthy throughout the experiment. After the experiment, swimming behavior of the animals was carefully observed to make sure of normal condition.

### Eye movement recording

Goldfish and carp eye movements were recorded using the scleral search coil technique^2,3,26^. Under local xylocaine anesthesia a 40-turn, 5-mm diameter insulated coil (IET Inc., Switzerland) was sutured at two points onto the upper scleral margin of both eyes with 8.0 ophthalmic silk. The water tank was placed in the center of a magnetic field (DNI inc., USA) generated by two sets of field coils driven by 2 sinusoids with different frequencies for the measurement of horizontal and vertical eye positions. Each of horizontal and vertical eye position signals from both eyes was digitized in synchrony with visual stimulation motion (see below). Horizontal eye position was calibrated by rotating the eye coil at the known angles in the field coil^2^.

Zebrafish and medaka eye movements were measured from video images^19,25^ acquired by an IR CMOS camera (DMK21BF04, THE IMAGING SOURCE). The camera with IR filter (IR-86, Fujifilm Corporation) placed atop the water tank acquired an image around the head of a fish illuminated with infrared light (AE-LED56, Akizuki Denshi Tsusho CO., LTD.) at 60 frames/s. The acquired images from the camera were sent to a PC via IEEE 1394 at 60 Hz, and monocular eye positions were estimated by custom-made software developed on LabVIEW Vision Toolkit (National Instruments). The images and the estimated eye position data were saved in the hard drive of the same PC with visual stimulus motion data (see below).

### Visual stimulation

In goldfish and carp experiments, visual stimulation was provided by a servo-controlled planetarium that could be rotated 360° around the vertical axis at speeds ranging from 0 to 100°/s constant velocity^2,3,26^. The planetarium projected a random light spot pattern on to the walls of the water tank. The rotational axis of the planetarium was carefully aligned to the center of the animal’s head at the level of the horizontal semicircular canals. Horizontal and vertical eye including planetarium positions were measured by a potentiometer (Midori Precisions Co., Ltd., Japan). The signals were continuously digitized at a sampling frequency of 1000 Hz in 16-bits with the use of a Power1401 interface (Cambridge Electronic Design, UK) for display and storage using the Spike2 program. The commands to drive the planetarium were also generated in Spike2 and D/A converted by Power1401.

In the zebrafish and medaka experimental setup, the visual stimulus was also a random light spots pattern projected on to the wall of the water tank by a planetarium placed under the transparent floor of the tank rotated by a servomotor (CM1-17L30A, MUSCLE Corporation) by using LabVIEW via a DAQ (NI USB-6009, NATIONAL INSTRUMENTS)^19^.

### Experimental paradigm

Thirteen adult goldfish, nine zebrafish, thirteen medaka, seven vestibular-neurectomized goldfish, and three carp were employed for the present experiments. The planetarium was rotated at a constant speed of 20 deg/s alternately in both clockwise (CW) and counter clockwise (CCW) directions for 8 s each^2,3^. This stimulus cycle was repeated for 30 (all species) or 180 minutes (goldfish and carp). OKAN in clockwise (CW) and counter-clockwise (CCW) directions were measured twice after exposure to a constant velocity (20 deg/s) visual stimulation for 1 min in all normal goldfish, five zebrafish, five medaka, three carp, and all (7) vestibular-neurectomized goldfish. The VOR in the dark during sinusoidal head rotation (amplitude: 40 deg/s, frequency: 0.125 Hz) was measured in vestibular-neurectomized goldfish to confirm completeness of the neurectomy.

### Surgical procedures

Before experimental sessions, goldfish and carp were anesthetized by immersion in a solution 1:20.000 wt/vol of MS222 (tricaine methanesulfonate, Sigma). A pedestal of dental acrylic cemented to self-tapping screws was fastened to the frontal bones to provide head stabilization during experiments^27^. In goldfish, a 3-mm triangle window was trephined in the occipital bone to allow access to the cerebellum or the vestibular nerves. The bone flap was reattached with cyanoacrylic glue and removed for cerebellectomy or vestibular neurectomy. For cerebellectomy, the cerebellum was aspirated in 1 ~ 3 minutes through a 23-gauge needle^3,23^. For vestibular-neurectomy, the superior branch of VIIIth nerve consisting of anterior and horizontal canals along with utricular fibers was sectioned bilaterally 1 ~ 3 days prior to experiments.

All relevant guidelines were followed for use and care of animals in the study, and the animal welfare committee of Chubu University approved all these experimental and surgical procedures (Approval ID: 3010066).

### Data analysis

Data recorded in Spike2 and LabVIEW were exported to MATLAB (Mathworks, USA), and all analyses were done offline in MATLAB. Eye velocity of goldfish and carp was calculated by applying a 3-point low pass differentiation filter to eye position data sampled at 1000 Hz. High frequency noise components amplified by the differentiation processing were eliminated by applying twice a 101-point moving average filter with a cut-off frequency of 4.39 Hz in offline analysis. The eye positions of zebrafish and medaka were resampled from 60 Hz to 100 Hz by using MATLAB interp1 function, and eye velocity was calculated by applying once a 3-point low-pass differentiation. High frequency noise components amplified by the differentiation processing were eliminated by applying a 11-point moving average filter twice with a cut-off frequency of 4.04 Hz in offline analysis. Different filters were used for data from different measurement systems so that their cutoff frequencies are close each other and enough to eliminate high frequency noise effectively from both data sets. Saccades and post-saccadic drifts, if present, were eliminated by applying a custom made automatic desaccading algorithm using an acceleration threshold^28^. In this study, the eliminated portions of the data were not used for any other analyses.

In order to evaluate predictive deceleration components of OKR eye velocity before stimulus direction changes, eye velocity traces were averaged over individual animals in each species during the training paradigm by alignment with the visual stimulus waveforms. Average values were calculated excluding the portions eliminated due to desaccading. Values of predictive deceleration during visual training were calculated from the average eye velocity waveforms for each stimulus cycle. In each half cycle of visual stimulation (8 s), average eye velocity data between 5.5 and 7.5 s was approximated by the following linear regression model:

1 $${e}_{OKR}(t-5.5)=a(t-5.5)+b+\epsilon (t-5.5),\,5.5 < t < 7.5$$

where *a* and *b* are the slope of eye velocity trace during (5.5 < *t* < 7.5) and the eye velocity value at *t* = 5.5, respectively, *t* [s] denotes time from the beginning of each stimulus half cycle, and *ε* is the residual. The estimated variable *a* for CW and CCW that minimizes the squared sum of the residual *ε* were averaged to obtain the deceleration value for the stimulus cycle.

To quantify OKAN duration, OKAN eye velocity traces measured twice in each of CW and CCW direction were averaged in each fish. The following decaying exponential model was fit to averaged OKAN eye velocity traces to estimate the time constant *τ* of OKAN:

2 $${e}_{OKAN}(t)=A+B\,\exp (\,-\,t/\tau )+\zeta (t),0 < t < 40$$

where *A*, *B* and *τ* are parameters to be estimated, and *t* [s] denotes time after the visual stimulation was turned off. The parameters minimizing the squared sum of residual *ζ* were estimated by using a non-linear optimization method in MATLAB (lsqnonlin).

## Results

### Predictive OKR in carp, zebrafish, and medaka compared with goldfish

#### General characteristics of predictive OKR in goldfish

Figure 1 summarized predictive OKR in goldfish (modified from Fig. 1 of Miki *et al*., 2018^3^). During bidirectional velocity step visual stimulation (Fig. 1A, gray line), left (L, orange line) and right (R, green line) conjugate changes in eye position consist of slow and fast phases (gray allows). At the beginning of the training (Control) slow phase eye velocity gradually builds-up after an initial rapid jump and approaches the visual stimulation velocity until the stimulation direction changes (black arrows). After bidirectional velocity step visual training for 3 h (Trained) eye velocity started downward before stimulus direction switched (black arrows). Averaged eye velocity traces over 8 goldfish illustrates more clearly this predictive OKR (Fig. 1B Training Stimulus Period, Trained).

When the stimulation period was extended twice as long as the training stimulation period after the acquisition of predictive OKR, eye velocity continued to decrease even though the visual stimulation was still moving in the same direction (Extended Stimulus Period, Trained). This predictive OKR in the Extended Stimulus Period usually continues more than 110 cycles after 3 h training. When the stimulation was turned off instead of being extended, the eye velocity profile during training continued in the dark for several stimulus cycles (Fig. 1C, Trained). These features were strong indications that the fishes learned the timing to predict when the direction of eye movement were to occur in response to temporally periodic visual perturbation generating OKR. To quantify the strength of predictive OKR we evaluated the slope of the line fitted to the deceleration part of eye velocity trace (Fig. 1B, Training Stimulus Period, dotted lines. Equation(1) in Methods). We refer to the slope “Predictive Deceleration” or just “Deceleration” in this study. Also, we refer to the rapid initial jump and the gradual build-up components constituting OKR eye velocity as “Initial Jump” and “Build-up”, respectively (Training Stimulus Period, thin black arrows).

### Predictive OKR in other fish species

**Figure 2:**
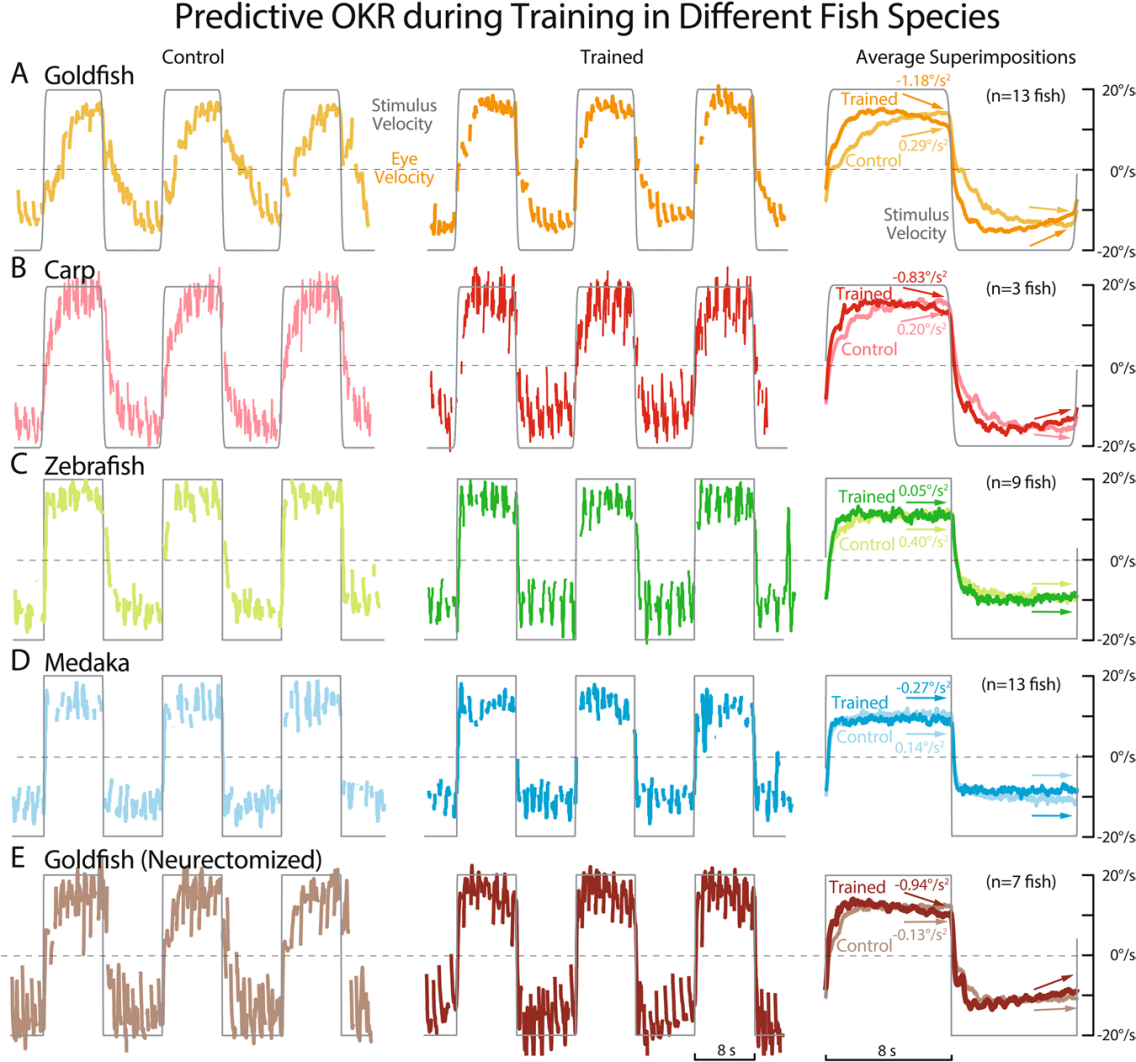
Predictive OKR during Training in Different Fish Species. Eye movement performance of a representative normal goldfish (**A**), carp (**B**), zebrafish (**C**), medaka (**D**) and vestibular neurectomized goldfish (**E**) at the beginning (left, Control) and the end (middle, Trained) of 30-min bidirectional velocity step training with half-stimulus period of 8 sec. Right panels show mean eye velocity traces of each fish group (normal goldfish n = 13, carp n = 3, zebrafish n = 9, medaka n = 13, neurectomized goldfish n = 7) averaged over the initial (Control, pale color) and the last (Trained, dark color) 18 stimulus cycles (for 5 min). Numbers by the arrows indicate mean Predictive Deceleration values.

Figure 2 shows eye velocity traces of a typical carp (B), zebrafish (C), and medaka (D) at the beginning (left panels, Control) and the end of bidirectional velocity step visual training for 30 min (middle panels, Trained), and superimpositions of their averaged traces over different animals in the same species (right panels) to compare with those of goldfish (A). For all species, Trained data were recorded at 30 min after the beginning of the visual training to assure that goldfish presented clear predictive OKR (A, Trained, dark orange arrows) while all animals were maintained healthy and active throughout the experiments. The same bidirectional periodic velocity step visual stimulation (gray lines, 8 s CW – 8 s CCW at 20 deg/s) was used for all animals as in Fig. 1. From eye velocity traces, fast phases were eliminated for a display purpose, as well as to calculate average traces.

Eye velocity of carps (Control, B, left & right panels, light red line) showed a small Initial Jump in response to an abrupt change in stimulus direction, followed by a Build-up component until the next stimulus direction switch. After the visual training, eye velocity (Trained, B, middle & right panels, dark red line) showed a greater Initial Jump and a faster Build-up than those in Control. These features were qualitatively similar to those of goldfish as exemplified in Figs. 2A and 1B, and as reported previously for OKR gain adaptation^2,29–31^. In addition, carps presented a Predictive Deceleration starting prior to the changes in stimulus direction (Trained eye velocity in Fig. 2B, middle panel), that was not present in Control records (B, left panel). This Predictive Deceleration can be seen more clearly in the averaged eye velocity traces (B, right panel, dark red arrows) in comparison with that in Controls (B, right panel, light red arrows). Mean Deceleration value of Control (0.20 deg/s^2^) and that of Trained (−0.83 deg/s^2^) are significantly different (t-test, p = 0.0015). For comparison, mean Deceleration values of goldfish in Control and Trained (Fig. 2A) are 0.29 deg/s^2^ and −1.18 deg/s^2^, respectively.

Zebrafish (Fig. 2C) and medaka (D), by contrast, did not show clear changes in their eye velocity profiles after the visual training, including Predictive Decelerations. Their mean Deceleration values in Trained (0.05 deg/s^2^ for zebrafish, and −0.27 deg/s^2^ for medaka) were not significantly different from those of Control (0.40 deg/s^2^ for zebrafish, 0.14 deg/s^2^ for medaka, t-test, p = 0.21 for zebrafish, p = 0.099 for medaka). Unlike goldfish and carp, averaged eye velocity of medaka in Control (D, right panel, light blue) presented large Initial Jump and fast Build-up that were unchanged in Trained averaged eye velocity (dark blue). Averaged eye velocity traces of zebrafish (C) were similar to those of medaka, with slightly greater Initial Jump and faster Build-up in Trained than those in Control. Also averaged eye velocities of both zebrafish and medaka are smaller than those of goldfish and carp. Previous study^2^ has shown in goldfish that predictive OKR occurs at much slower eye velocity (4 deg/s), suggesting that slower eye velocity in zebrafish and medaka is not a factor of their inability to acquire Predictive Decelerations.

**Figure 3:**
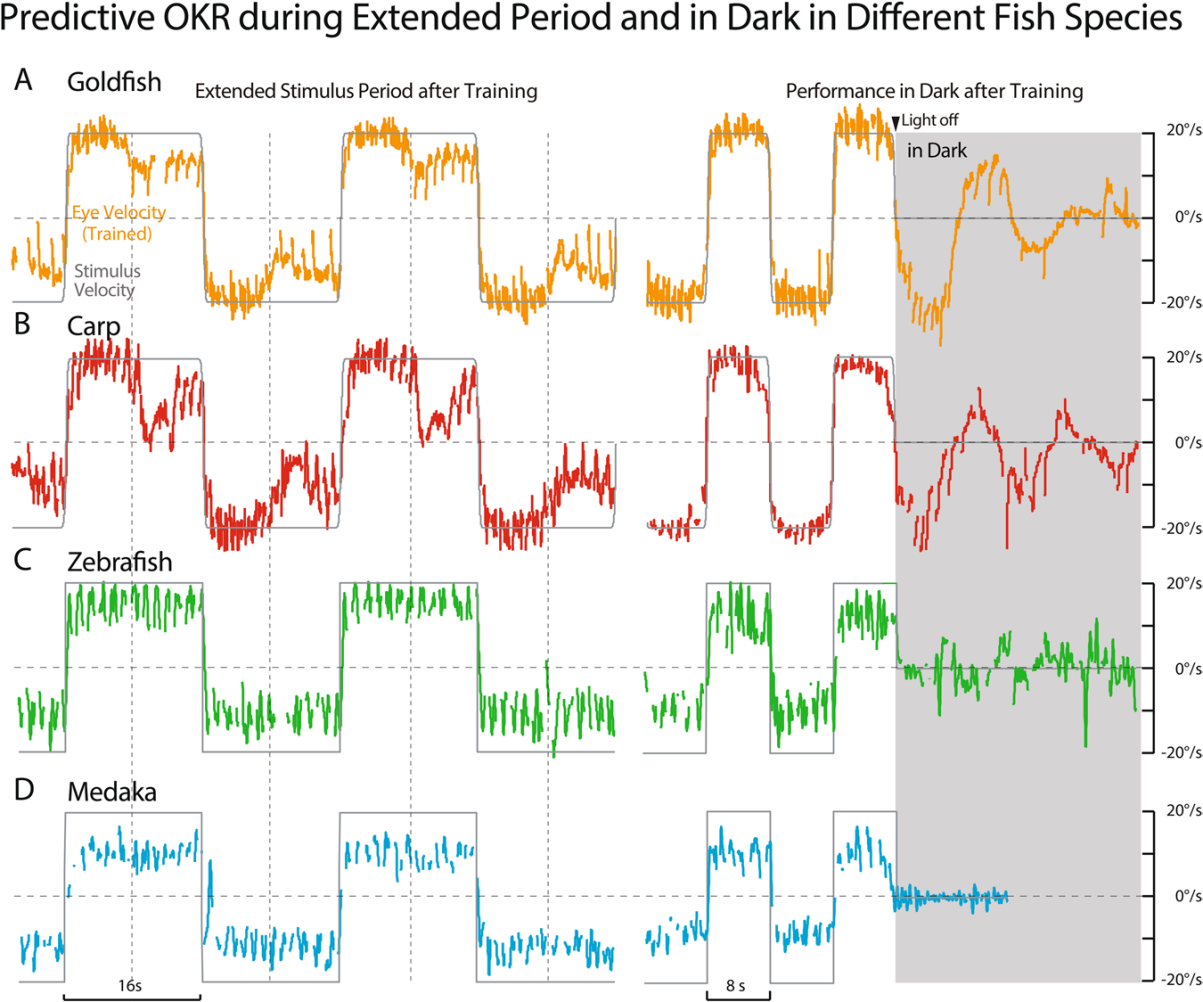
Predictive OKR during Extended Period and in Dark in Different Fish Species. Eye movement performance during extended period stimulation with a half-period of 16 sec (Left) and in the dark (Right) after bidirectional velocity step training whose half-period was 8 sec in representative goldfish (**A**), carp (**B**), zebrafish (**C**), and medaka (**D**).

Figure 3 shows eye velocity traces of representative fish in each species during Extended Stimulus Period (left panels) and in Dark (right panels) after the same visual training as in Fig. 2. Carp (B) presented clear Predictive Deceleration during Extended Stimulus Period (half cycle 16 s), and eye velocity oscillation in dark at the training period (half cycle 8 s) as those in goldfish (A). By contrast, none of zebrafish and medaka tested presented any of these clear indications of predictive OKR as exemplified in C and D, respectively. These results shown in Figs. 2 and 3 demonstrate that carps acquired predictive OKR like goldfish while zebrafish and medaka scarcely did.

### Optokinetic after nystagmus (OKAN) in goldfish, carp, zebrafish, and medaka

**Figure 4:**
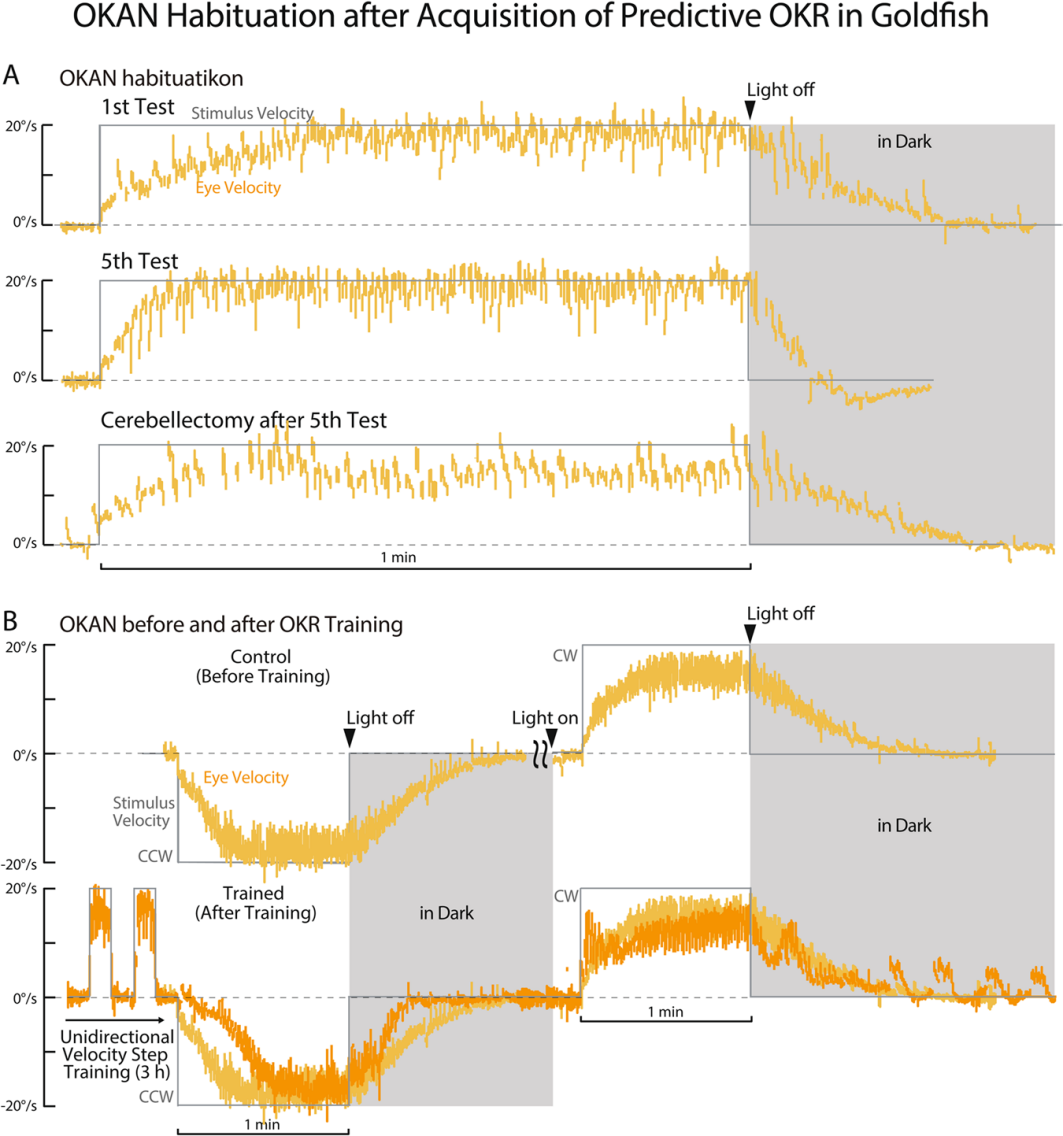
OKAN Habituation after Acquisition of Predictive OKR in Goldfish. (**A**) OKAN of a representative goldfish after 1^st^ (top) and 5^th^ (middle) constant velocity visual stimulation for 1 min. The bottom trace shows OKAN of the same goldfish after acute cerebellectomy after the 5^th^ test. (**B**) OKAN tested before (top) and after (bottom) unidirectional (clock-wise) velocity step training^3^ for 3 hours in a representative goldfish.

OKAN is a continuing eye velocity in the dark after a prolonged constant velocity visual stimulation (See Methods), and its slow phase eye velocity is well approximated by a decaying exponential function as in Eq. (2). OKAN has been observed to habituate^2,32,33^, wherein duration gets shorter when OKAN is repeatedly measured as exemplified in Fig. 4A. OKAN of a typical goldfish in the first measurement lasted longer than 20 sec (1st Test), while that in the 5th measurement lasted less than 10 sec (5th Test). Notably, not only OKAN, but also the time constant of Build-up component at the onset of the constant velocity visual stimulation shortened in the 5th test. Acute cerebellectomy after the 5th test that took about 2 min^3^, de-habituated OKAN duration and returned the Build-up time constant back to values comparable to those in the 1st test (Cerebellectomy after 5th Test). In addition, cerebellectomized OKAN never habituated (data not shown).

OKAN after acquisition of predictive OKR in goldfish was also habituated (Fig. 4B). This figure illustrates OKAN measurements following each of CCW and CW constant velocity 20 deg/s visual stimulation for 1 min in a typical goldfish before (B, Control) and after (B, Trained) unidirectional (CW) velocity step visual training for 3 h^3^. Before the visual training OKAN following both CW and CCW steps lasted longer than 50 s (B, Control). OKAN immediately after unidirectional (CW) visual training was habituated with a duration less than 20 s in the untrained direction (CCW). For comparison the eye velocity trace of Control (B, light orange) was superimposed on that of Trained (B, dark orange). Evaluation of OKAN in the trained direction (CW) after unidirectional visual training was difficult due to the oscillatory eye velocity as the training period continued in the dark (cf. Figure 1C).

**Figure 5:**
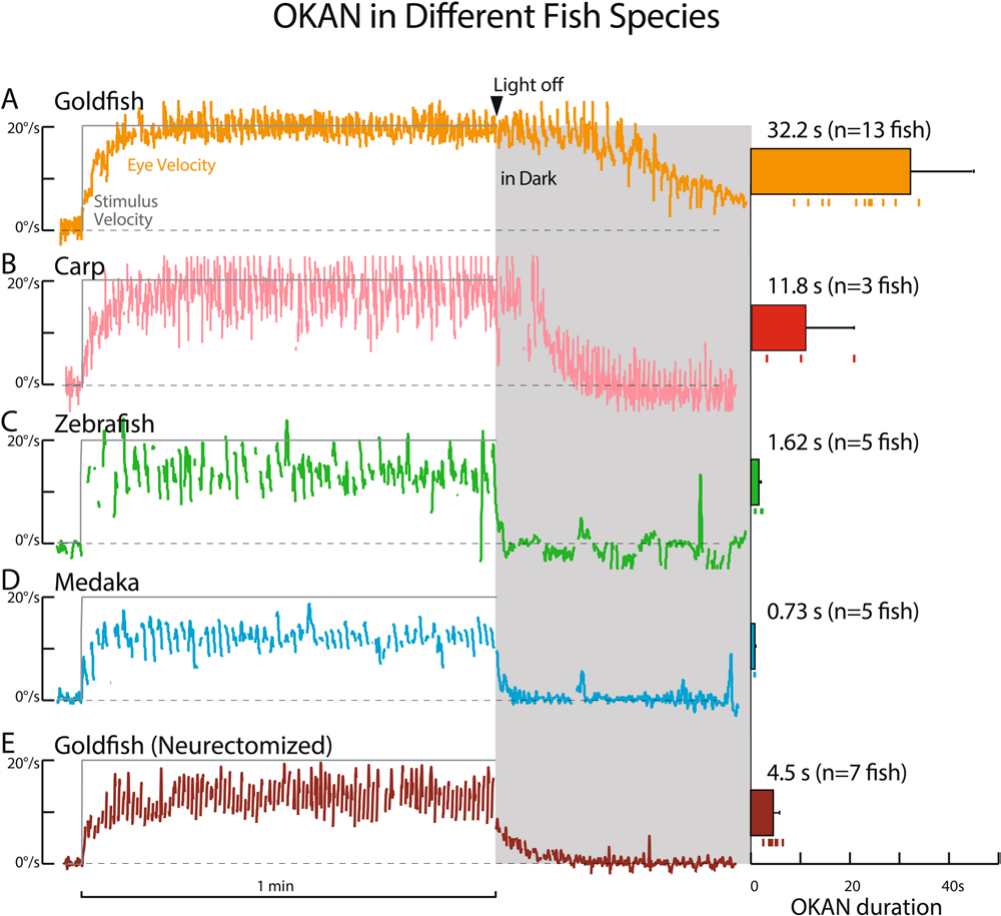
OKAN in Different Fish Species. OKAN of a representative normal goldfish (**A**), carp (**B**), zebrafish (**C**), medaka (**D**), and neurectomized goldfish (**E**) along with mean OKAN duration in each fish species (right). Error bars are standard deviation. OKAN durations of goldfish (both normal and neurectomized) and carp are time constant *τ* [s] in Eq. (2) in Methods while those of zebrafish and medaka are time [s] until eye velocity became slower than 2 deg/s after stimulus light was turned off, because OKANs were too short to approximate and estimate *τ* with Eq. (2).

Figure 5 compares OKANs in goldfish (A), carp (B), zebrafish (C), and medaka (D). OKANs were measured before visual training from the same animals used in Figs. 2 and 3. Identical visual stimulation at a constant velocity (20 deg/s) was given to each fish for 1 min and then the stimulus was turned off to observe OKAN in the dark. As exemplified in Fig. 5A and B, respectively, goldfish and carp showed robust long-lasting OKAN. By contrast zebrafish (C) and medaka (D) hardly exhibited OKAN. Averaged OKAN duration over the same fish species were 32.2, 11.8, 1.62, and 0.73 s for goldfish (n = 13), carp (n = 3), zebrafish (n = 5), and medaka (n = 5), respectively (Fig. 5, right panels). Thus, goldfish and carp that acquired predictive OKR exhibited robust OKAN, while zebrafish and medaka that did not acquire clear predictive OKR all showed very short OKANs. Notably the standard deviation of goldfish OKAN duration time (Fig. 5A, right panel, error bar) was quite large (see below).

### Relationship between strength of prediction and OKAN duration

#### Normal animals

Zebrafish and medaka which presented very short, if any, OKAN did not acquire predictive OKR while goldfish and carp exhibited long OKAN with predictive OKR. These results suggest that OKAN duration could be an important factor for determining predictive OKR with those fish exhibiting longer OKAN acquiring predictive OKR better. All the normal goldfish tested (n = 13) showed robust OKAN distributed with a large variation from a maximum of 99 s to a minimum of 9 s with a median of 32 s (Standard deviation of 25 s). We therefore evaluated the strength of acquired predictive OKR as Predictive Deceleration in relation to OKAN duration among individual goldfish.

**Figure 6:**
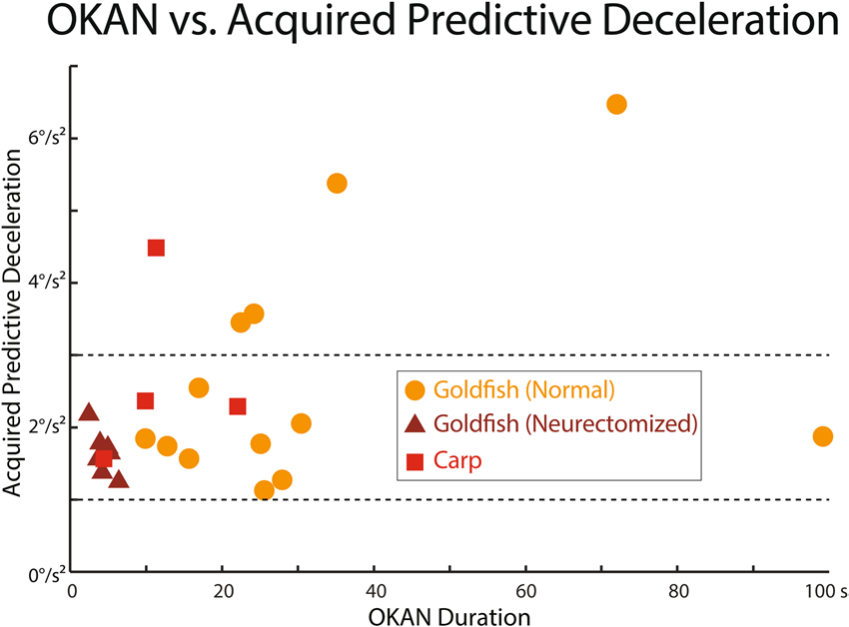
OKAN duration vs. Acquired Predictive Deceleration of individual fish. Orange: normal goldfish (n = 13), red: carp (n = 3), brown: neurectomized goldfish (n = 7). Predictive Decelerations were measured from averaged eye velocity traces over the last 25 stimulus periods of 3-h bidirectional visual training. OKANs were measured before visual training, and time constants *τ* were estimated by using Eq. (2) in Methods.

Figure 6 illustrates the relationship between OKAN duration (*τ* in Eq. (2) in Methods) and Predictive Deceleration after 3 h visual training for individual fish. We employed Trained data after 3 h instead of 30 min as in Fig. 2 to evaluate a fully adapted state of each animal. Each filled orange circle represents data from each of the 13 normal goldfish. Notably, there was only a slight positive correlation (r = 0.36) between the two values without statistical significance (t-test, p = 0.22). For comparison, data from individual carps used in Fig. 2B were trained for 3 h and the results plotted as red filled squares in Fig. 6. These plots intermingled with those of goldfish, and did not show a clear correlation between OKAN duration and acquired Predictive Deceleration. Together these results suggest that animals with longer OKAN do not necessarily acquire better predictive OKR.

#### Vestibular-neurectomized animals

**Figure 7:**
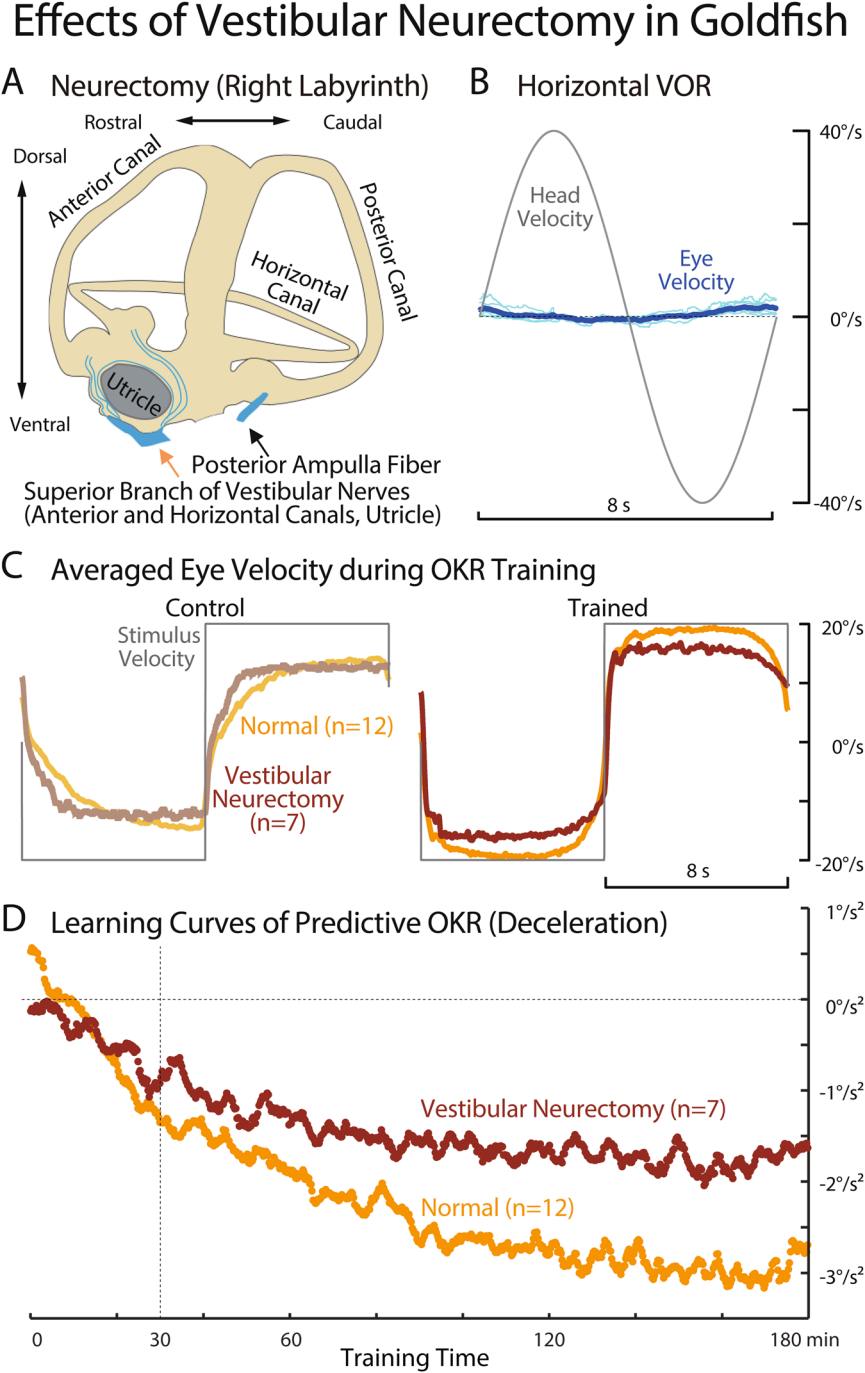
Effects of Vestibular Neurectomy in Goldfish. (**A**) Schematics of right semi-circular canals and sectioned vestibular nerves. Vestibular neurectomy was done bilaterally. (**B**) Horizontal vestibuloocular reflex measured in the dark in response to a sinusoidal head velocity stimulation at 0.125 Hz with the amplitude of 40 deg/s. Thin cyan lines indicate eye velocity traces during each of 30 stimulus cycles, and thick blue lines indicates their average. (**C**) Comparison of averaged eye velocity traces just after (Control) and 3 hours after (Trained) the beginning of bilateral velocity step visual training (gray) between normal (pale and dark orange traces) and vestibular neurectomized goldfish (pale and dark brown traces). (**D**) Learning curves of predictive deceleration of normal (orange) and vestibular neurectomized goldfish (brown). Predictive decelerations (*a* in Eq. (1) in Methods) were estimated from averaged eye velocity traces over 13 fish (normal) and 7 fish (neurectomized) for each stimulus cycle. A moving average spanning 11 stimulus cycles was applied to both learning curves to reduce variabilities in the estimation of predictive deceleration.

Vestibular-neurectomy has been shown to markedly shorten OKAN duration^32,34,35^. In order to directly explore the uncorrelated relationship between predictive OKR ability and OKAN duration in the same experimental species, VIIIth nerve neurectomy was carried out in goldfish. The superior branch of VIIIth nerve containing the horizontal and anterior canal afferents as well as those from utricle was cut bilaterally at least 1 ~ 3 days before predictive OKR experiments (Fig. 7A, orange arrow). In their home water tanks, neurectomized fish maintained normal postures although swimming behaviors were unstable. The horizontal VORs of the neurectomized fish measured before visual training were nearly absent (VOR gain: 0.012 +/− 0.015) as shown in Fig. 7B, confirming that horizontal semicircular canal afferents were effectively disconnected.

Figure 5E illustrates the OKAN of a typical neurectomized goldfish measured before visual training in comparison with normal goldfish (A) and other fish species (B-D). In contrast to intact goldfish (A), the OKAN of the representative neurectomized goldfish lasted significantly shorter as previously demonstrated in goldfish^36^ and other animal species^32,34^. Mean OKAN duration for 7 neurectomized goldfish was 4.5 s (Fig. 5E, right panel) in contrast to 32.2 s for normal animals. The variability of OKAN duration time in neurectomized animals (SD: 1.2 s) was much smaller than that in the normal group (SD: 25 s).

Figure 2E illustrates a typical horizontal eye velocity trace of a vestibular neurectomized goldfish recorded just after (Control, left panel) and 30 min after (Trained, middle panel) the beginning of the same visual stimulation (gray) as used for other fish in Fig. 2. Neurectomized goldfish generated seemingly normal OKR eye velocity with greater Initial Jump and faster Build-up than the normal animals (A, left panel). After 30 min of visual training (E, Trained, middle panel), eye velocity started to decrease before changes in the stimulus direction. This was also clearly seen in the averaged eye velocity traces over 7 neurectomized fish (E, right panel) in Control (right brown line) and in Trained (dark brown line). The averaged Trained eye velocity clearly started to slow down before changes in the stimulus direction (dark brown arrows). Predictive Deceleration values for Control and Trained are −0.13 deg/s^2^ and −0.94 deg/s^2^, respectively, and the difference is statistically significant (t-test, p = 0.0041).

In order to further evaluate differences in predictive OKR between normal and neurectomized animals in a fully adapted state, we compared in Fig. 7C averaged eye velocity traces at the beginning (Control) and 3 h after the visual training (Trained). Averaged eye velocity of neurectomized animal (Control, light brown) arose (Initial Jump) more rapidly than that of normal animal, and reached a plateau level slightly slower than the maximum velocity of the normal animals in both CW and CCW stimulus rotations. After 3 h of visual training, Trained eye velocity of normal animals arose more rapidly (greater Initial Jump), reached a greater maximum velocity than Control eye velocity, and began to slow down before changes in the visual stimulus direction as described in Figs. 1B, 2A and a previous study^3^. Similarly, Trained eye velocity of neurectomized animal jumped-up more rapidly, reached a greater maximum velocity than Control, and slowed down prior to changes in the visual stimulus directions. Namely, neurectomized goldfish acquired predictive OKR as in normal animals although on average Predictive Deceleration appeared smaller than in normal fish.

Figure 7D compares the averaged time courses of Predictive Deceleration for neurectomized goldfish (brown, n = 7) and normal animals (orange, n = 13). The orange trace for normal fish started from a positive value at the beginning of the visual training (Control) and decreased to negative, eventually reaching a plateau around −3 deg/s^2^ after 3 h. By contrast, the time course for neurectomized animals started near 0 and gradually decreased more negatively at a slower rate than the normal animals. Eventually a plateau was reached around −2 deg/s^2^ after 3 h.

To compare the relationship between OKAN duration and acquired Predictive Deceleration with that of normal goldfish, data from each of 7 neurectomized goldfish were superimposed in Fig. 6 (brown filled triangles). As noted above, OKAN durations were significantly shorter and less variable than those of normal animals with data presented on the left side along the abscissa. The neurectomized data are scattered in the region between 1 and 3 deg/s^2^ on the ordinate (horizontal dotted lines) in which reside data from 9/13 normal goldfish and 2/3 carp. These plots showed that neurectomized goldfish with OKAN duration severally shortened acquired predictive OKR comparable to about 70% of the normal goldfish population.

## Discussion

OKR has been observed in most vertebrate species and extensively studied to understand cerebellar dependent motor learning during OKR gain adaptation^29–31^. Predictive OKR was first recognized in goldfish^2^ and has been considered as a model system to understand neural mechanism of predictive motor control. As in any other predictive motor control system, the OKR benefits from counting the passage of time to precisely know when to move/stop the eyes ahead of changes in periodic visual motion. The cerebellum has been implicated to be involved in a neural representation of time that can be utilized for various tasks requiring precise timing^37^. Recent studies in monkeys performing a self-timed oculomotor task supported this notion, demonstrating that neurons in cerebellar dentate nucleus contribute to the fine tuning of self-timing^38,39^.

In goldfish the cerebellum was recently demonstrated to be necessary for acquiring predictive OKR^3^. In view of this finding, we tested the hypothesis that if the cerebellum alone was sufficient for the acquisition of predictive OKR then the proposed conserved basic cerebellar neuronal circuitry among vertebrate should extend, in particular, to closely related fish species^4–21^. However, we found that while carp and goldfish acquired predictive OKR behavior, zebrafish and medaka did not. (Figs. 2 and 3).

Interestingly, a previous study showed that zebrafish acquired a predictive optomotor behavior including correlated neural ensemble activity in the optic tectum^40^. The rhythmic visuo-motor transformations involved extended over a comparable time interval (i.e., seconds) to the predictive OKR behavior currently evaluated here. Therefore, these contrasting observations suggested that acquisition of predictive OKR utilized a neural basis additional to, or different from, the predictive optomotor behavior.

Our findings showed that the two species, goldfish and carp, acquiring predictive OKR exhibited robust optokinetic after nystagmus (OKAN) with variable duration. However, the OKAN of zebrafish and medaka was extremely short, if at all (Fig. 5) and predictive OKR was not acquired. In goldfish and carp, eye velocity oscillation in the dark around the period of acquired predictive OKR (Fig. 3) was similar to OKAN in that both represent a continuum of eye velocity in the dark after prolonged visual stimulation. Together these results suggest that neuronal circuitry generating OKAN, called the velocity storage mechanism (VSM), might be a possible determinant for acquisition of predictive OKR.

Paradoxically though, we found that goldfish and carp that presented longer OKAN did not necessarily acquire predictive OKR better (Fig. 6). Furthermore, vestibular neurectomized goldfish whose OKAN was severely shortened to values closer to zebrafish/medaka still acquired predictive OKR as normal animals (Figs. 2, 6, 7). Together, these results suggest that OKAN duration when considered as capacity of the VSM does not determine the ability to acquire predictive OKR. It is thus likely that the existence of a neuronal mechanism within the VSM circuitry, irrespective of its capacity, is crucial for the acquisition of predictive OKR.

Theoretically, the VSM is a leaky integrator that processes head motion signal from the vestibular semi-circular canals providing acceleration-like head motion information to obtain better estimation of head velocity^41^. When velocity step visual stimulation is applied, the eye velocity signal is gradually charged in the VSM, and after the visual stimulation is suddenly turned off, the stored eye velocity signal gradually decreases as OKAN until the VSM is completely discharged.

Neuronal circuitry subserving the VSM has been postulated to lie within the brainstem vestibular nuclei^42^. A subgroup of neurons in the medial and superior vestibular nuclei in monkeys showed highly correlated firing modulation with OKAN eye velocity^43–45^. Electrical stimulation in this area modulate the VSM^46^ while midline sections of the commissural connection between bilateral vestibular nuclei abolished velocity storage capability^47^. The degenerated neurons following midline sections were found to be GABA_b_-ergic^42,48^, suggesting that mutual inhibitory connections between bilateral vestibular nuclei were a key element to form the VSM^46^.

**Figure 8:**
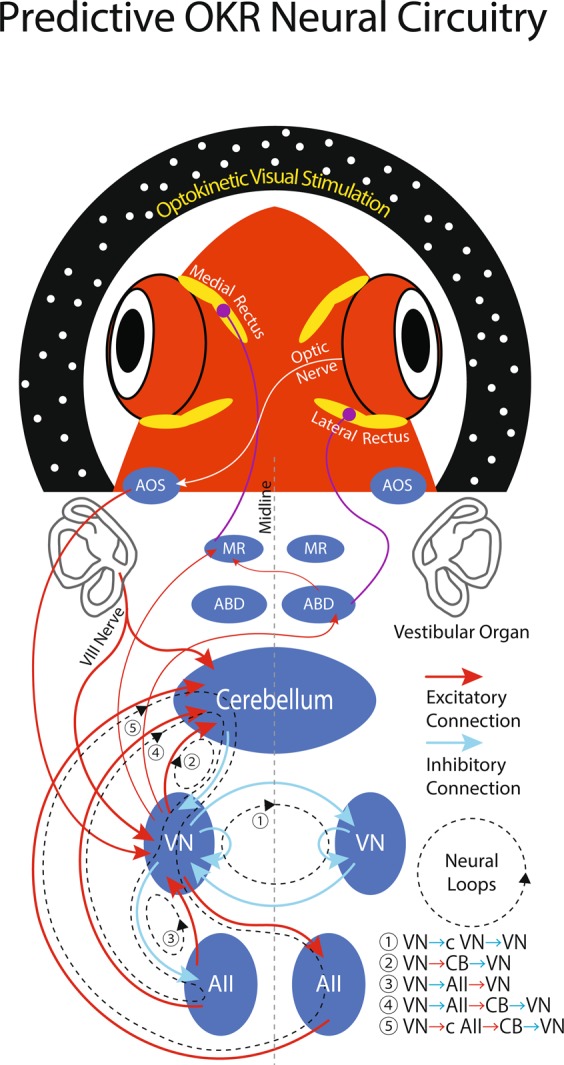
Predictive OKR Neural Circuitry. Experimentally established neuronal circuitry subserving horizontal OKR in goldfish^49–51^. For simplicity, only the connections on the left side of the brain are illustrated except for the bilateral commissural connections. Red and blue lines with arrow heads indicate excitatory and inhibitory connections, respectively. Black dotted lines labeled with ① ~ ⑤ indicate recursive neuronal connectivity (loops). VN: vestibular nucleus, AII: area II, ABD: abducens nucleus, MR: oculomotor nucleus innervating medial rectus, AOS: accessory optic system.

Similarly, in goldfish, it was recently reported that vestibular neurons in descending octaval nucleus (DO) show highly correlated firing modulation with OKAN eye velocity^49^. Neurons exhibiting firing modulation during OKAN have been found in Area II of goldfish as well^50^. These neurons in the DO and Area II have been identified to project to the cerebellum^51^ as illustrated in Fig. 8 summarizing the goldfish OKR neuronal circuitry^49–51^. Thus, the VSM signal is available in the cerebellum. The current results demonstrated that cerebellectomy did not abolish OKAN, but dishabituated the habituated OKAN (Fig. 4A). These results suggest that the cerebellum is not prerequisite for the VSM, but its output influences the VSM signal content via the inhibitory projection from the cerebellum to the VN (Fig. 8, cyan arrow forming the loops ②, ④, and ⑤). Thus, the recursive neuronal loops connecting bi-directionally the VSM and the cerebellum would enable the cerebellum to produce predictive motor command ahead of directional changes in the visual stimulation by utilizing the signal from the VSM that then is subsequently adjusted properly by the cerebellum to count passage of time.

In conclusion, our current results in the context of the well-defined neuronal connectivity of the goldfish OKR support our previous argument^3^ that the acquired predictive eye velocity behaviors appear to be dependent on feedforward VSM signals from the brainstem to the cerebellum, but the adaptive timing mechanism, itself, originates within the cerebellar circuitry. Namely, an internal model is formed that predicts future sensory stimulation from the past sequence of sensory input to generate oculomotor commands. Consequently the eye movement profile is continuously updated within the cerebellar neuronal circuitry by neural loops first employing, and then replacing the returning pre-cerebellar VSM signals arising from the brainstem. The computational algorithm and its neural implementation for this functionality are yet to be addressed by mathematical modeling^26^ and then tested by further behavioral and neurophysiological approaches.

## Data Availability

Data is available from the corresponding author upon reasonable request.
